# Technical report: surgical preparation of human brain tissue for clinical and basic research

**DOI:** 10.1007/s00701-023-05611-9

**Published:** 2023-05-06

**Authors:** J. Straehle, V. M. Ravi, D.H. Heiland, C. Galanis, M. Lenz, Junyi Zhang, N.N Neidert, A. El Rahal, I. Vasilikos, P. Kellmeyer, C. Scheiwe, J.H. Klingler, C. Fung, A. Vlachos, J. Beck, O. Schnell

**Affiliations:** 1 Department of Neurosurgery, Medical Center and Faculty of Medicine, University of Freiburg, Freiburg, Germany; 2 Department of Neuroanatomy, Institute of Anatomy and Cell Biology, Faculty of Medicine, University of Freiburg, Freiburg, Germany; 3 Center for Advanced Surgical Tissue Analysis (CAST), Faculty of Medicine, University of Freiburg, Freiburg, Germany; 4 Freiburg Institute of Advanced Studies (FRIAS), Freiburg, Germany; 5 Department of Neurological Surgery, Lou and Jean Malnati Brain Tumor Institute, Robert H. Lurie Comprehensive Cancer Center, Feinberg School of Medicine, Northwestern University, Chicago, Illinois USA; 6 Center Brain Links - Brain Tools, University of Freiburg, Freiburg, Germany; 7 Center for Basics in Neuromodulation (NeuroModulBasics), Faculty of Medicine, University of Freiburg, Freiburg, Germany

**Keywords:** Cortical access tissue, Human, Research, Neurosurgery, Tumor, Epilepsy

## Abstract

**Background:**

The study of the distinct structure and function of the human central nervous system, both in healthy and diseased states, is becoming increasingly significant in the field of neuroscience. Typically, cortical and subcortical tissue is discarded during surgeries for tumors and epilepsy. Yet, there is a strong encouragement to utilize this tissue for clinical and basic research in humans. Here, we describe the technical aspects of the microdissection and immediate handling of viable human cortical access tissue for basic and clinical research, highlighting the measures needed to be taken in the operating room to ensure standardized procedures and optimal experimental results.

**Methods:**

In multiple rounds of experiments (*n* = 36), we developed and refined surgical principles for the removal of cortical access tissue. The specimens were immediately immersed in cold carbogenated N-methyl-D-glucamine-based artificial cerebrospinal fluid for electrophysiology and electron microscopy experiments or specialized hibernation medium for organotypic slice cultures.

**Results:**

The surgical principles of brain tissue microdissection were (1) rapid preparation (<1 min), (2) maintenance of the cortical axis, (3) minimization of mechanical trauma to sample, (4) use of pointed scalpel blade, (5) avoidance of cauterization and blunt preparation, (6) constant irrigation, and (7) retrieval of the sample without the use of forceps or suction. After a single round of introduction to these principles, multiple surgeons adopted the technique for samples with a minimal dimension of 5 mm spanning all cortical layers and subcortical white matter. Small samples (5–7 mm) were ideal for acute slice preparation and electrophysiology. No adverse events from sample resection were observed.

**Conclusion:**

The microdissection technique of human cortical access tissue is safe and easily adoptable into the routine of neurosurgical procedures. The standardized and reliable surgical extraction of human brain tissue lays the foundation for human-to-human translational research on human brain tissue.

**Supplementary Information:**

The online version contains supplementary material available at 10.1007/s00701-023-05611-9.

## Introduction

Access to human brain tissue is limited, and sampling for research faces technical and ethical challenges. There is limited information available on patient consent, as well as technical and logistic requirements for neurosurgeons, technical staff, and scientists to conduct research using human brain tissue. The neurosurgeon responsible for tissue removal primarily has to guarantee patient safety and the success of the procedure. However, in cases of deep-seated pathologies, cortical and subcortical access tissue, which is often removed and discarded, could be dissected and used to analyze human-relevant functional, structural, and molecular mechanisms in health and disease [2, 6, 16].

**Fig. 1:**
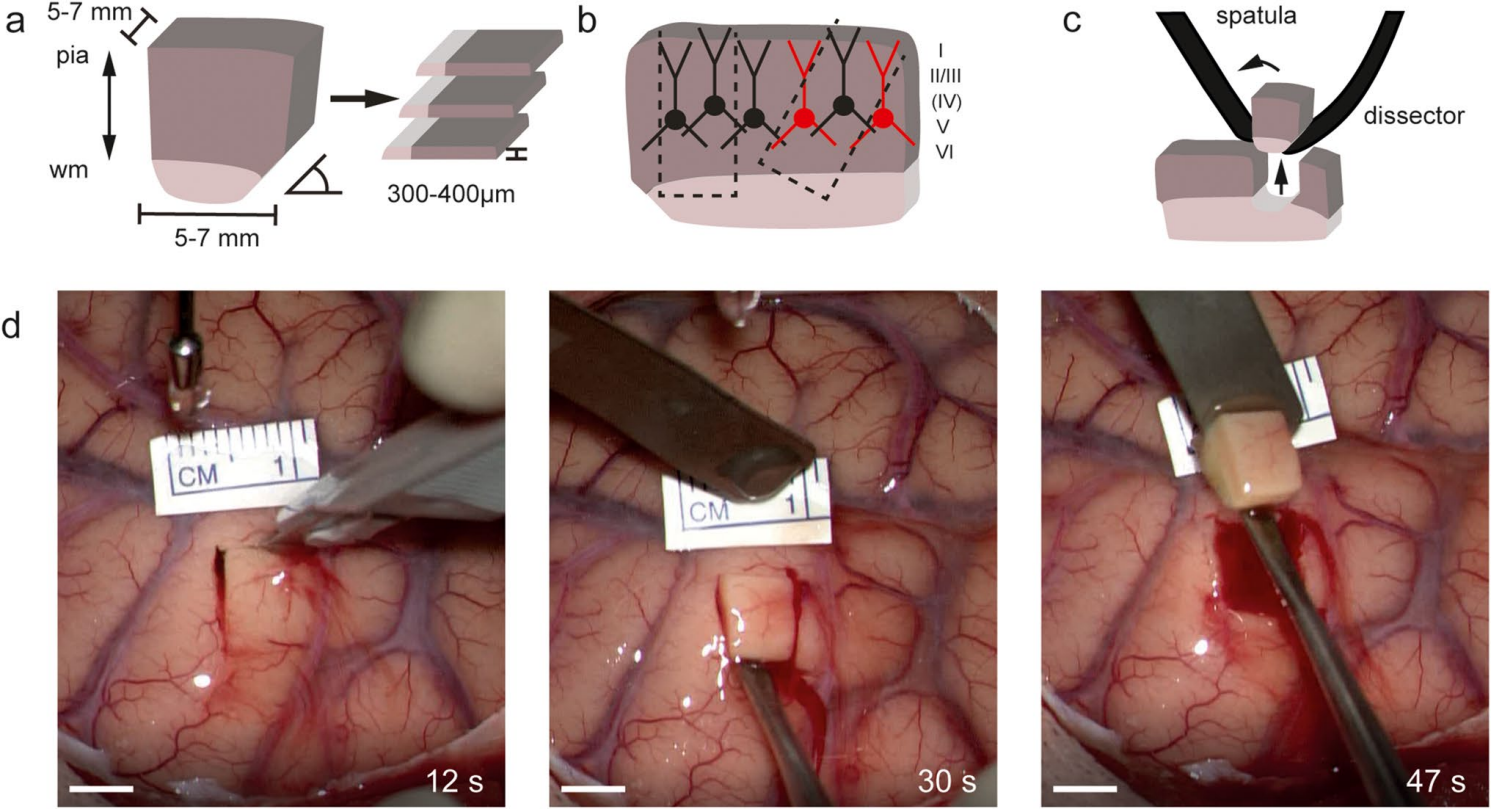
Illustration of the preparation of small samples according to the principles of tissue microdissection (Table 1). (**a**) Dimensions of a small cortical tissue sample which will be cut into brain slices in the laboratory. Pia mater and wm (white matter). (**b**) Illustration of damage of cortical neurons when the cortical axis is not preserved during sample microdissection. (**c**) Illustration of technique to retrieve sample with minimal damage to sample and surrounding cortex. (**d**) Intraoperative images of the technique of microdissection corresponding to case 1 (Fig. 3). Scale bar 5 mm. Lower right indication of time (s) after the onset of preparation

For experiments with vital brain tissue, it is necessary to prepare acute brain slices with a thickness of 300 to 400 μm using a vibrating microtome (vibratome) (Fig. 1a). Slicing allows the tissue that has been severed from circulation to receive glucose and oxygen through diffusion. Different methods for acute brain slice preparations and organotypic slice cultures of human tissue have been described [9, 23, 38]. Using these methods, cortical access tissue was successfully used for electrophysiological experiments ex vivo [10, 12, 19, 40] including the investigation of the effects of pharmaceuticals at a cellular and synaptic level [15].

Volumetric electron microscopy studies of the human cortex obtained from neurosurgical procedures have also been recently reported [16, 32]. Such studies highly depend on tissue quality and preservation. Various fixation protocols have been described such as block immersion fixation [32, 42], preparation of acute slices in fixative [16], or fixation of acute tissue slices after recovery [15, 41].

Organotypic human brain tissue cultures can be successfully cultured for several weeks [1, 14, 23, 31, 37]. This methodological advancement opens a range of possibilities also for long-term experiments including neurooncological studies involving tumor growth, the tumor micromilieu, immunological responses, and pharmaceutical treatment ex vivo [7]. This, in turn, may allow for more personalized approaches to neurooncological treatment.

Although optimal tissue preservation is the key to standardization of sample quality allowing reproducible experimental investigations, a description of the surgical technique of how to safely and optimally microdissect and remove cortical access tissue is still lacking. Here, we describe the surgical technique and technical aspects of microdissection and immediate treatment of human cortical access tissue for basic and clinical research, highlighting the measures needed to be taken in the operating room (OR) to ensure optimal experimental results, thereby placing the neurosurgical OR at the center of neurooncological and neurophysiological studies.

## Methods

All human tissue samples were microdissected and processed with the approval of the local ethics committee (Biobank of the Department of Neurosurgery and Institute of Neuropathology, AZ 472/15_160880, AZ 593/19 and AZ 100020/09). Written informed consent was obtained from all patients. Tissue collection was performed from July 2019 to March 2021. The tissue samples were obtained as part of clinically indicated neurosurgical operations and were not resected for the purpose of research only.

As part of the preoperative planning, a safe putative cortical entry point was defined. Intraoperatively, after exposure of the pial surface, each surgeon evaluated and, if necessary, adjusted the cortical access according to criteria including macroscopic anatomy, position of superficial blood vessels, neuronavigation (Cranial Map Neuro Navigation Cart 2; Stryker), and where applicable intraoperative ultrasound (BK Medical). The location of the cortical access tissue was saved in the neuronavigation software for post-hoc analysis. Next, in contrast to standard neurosurgical training, the cortical access tissue was not cauterized, aspirated, or removed and discarded, but recovered for scientific investigations. In multiple rounds of experiments including feedback from scientists, the surgical technique of tissue dissection was iteratively developed and refined. Tissue was cut using a pointed scalpel (no. 11; Feather, Japan), disconnected from the white matter using a dissector (OL165R; Aesculap Surgical), and recovered using a brain spatula.

### Transportation media

For electrophysiological experiments, cold (7–10°C) N-methyl-D-glucamine-based artificial cerebrospinal fluid (aCSF) saturated with carbogen (95% O_2_, 5% CO_2_) was used as described in the literature [15, 38]. Larger (10–30 mm) samples used for organotypic slice cultures and transcriptomic experiments were transferred to sterile ice-cold carbogenated “preparation medium” (Hibernate-A Medium (Gibco) supplemented with 1 mM GlutaMAX (Gibco), 13 mM Glucose (Sigma-Aldrich), 30 mM NMDG (Sigma-Aldrich), and 1% Anti-Anti). Depending on the distance from the OR to the laboratory facilities, the transport time ranged from 5 to 15 min. For a transport time >5 min, the medium was carbogenated using a nitrile examination glove filled with carbogen (95% O_2_, 5% CO_2_) and connected to a 3-way valve (Luer/Lock) and perfusor line (B. Braun Melsungen AG) (Supplementary Fig. 1). A single glove (size XL) lasted up to 30 min.

### Patch-clamp recordings and visualization of neurons

The protocols for the recovery of acute human brain slices, electrophysiological whole-cell patch-clamp recordings, post-hoc visualization of neurons, and immunohistochemistry were performed as previously described [15]. Light microscopic images were acquired using a Leica SP8 laser-scanning confocal microscope equipped with a 40× oil-immersion (NA 1.30; Leica) objective.

### Electron microscopy

Acute 400 μm slices were immersed in EM fixative (4% Paraformaldehyd (Polyscience) (w/v) 2.5% Glutaraldehyd (Serva) (w/v) in 0.1 M phosphate buffer) and kept at room temperature for 2 h, then stored at 4 °C. Next, ~1.5 mm wide samples were trimmed spanning the entire cortical height. Staining and embedding of samples was then performed as described in the literature [15]. Electron micrographs were taken with a Philips CM100 transmission electron microscope equipped with a Gatan Kamera Orius SC600 with a magnification of 6600.

### Processing of large brain tissue sample

After transport of the samples to the laboratory, they were further divided into blocks ranging from 1 to 40 mm depending on the experimental use case (Table 2). Dissection was performed using sterile tools on a cutting platform consisting of aseptic filter paper (Whatman Merck, UK) moistened with sterile “preparation medium” (see above) and placed on an ice-cold metal block (−20 °C) on a sterile surgical drape. Visibly damaged or pathological tissue and capillaries were removed. For slice culture experiments, tissue blocks (7–20 mm) were cut into 300-μm-thick sections in a disinfected vibratome (Leica VT1200 Wetzlar, Germany) and filled with cold, sterile carbonated preparation medium. Depending on the apparent stiffness of the tissue, the cutting speed ranged from 0.14 to 0.16 mm/s with an amplitude of 1.5 mm. Immunofluorescent labeling was performed, and ex vivo glioblastoma invasion models were generated as described in the literature [7, 23] by injecting primary cultured GBM cells that were virally tagged with zsGreen into human organotypic slice cultures.

### Stimulated Raman histology

Small 1–4 mm large tissue and tumor samples were removed using a 2-mm cutting forceps (8591A; Karl Storz, Tuttlingen, Germany). The samples were then compressed in a custom microscope slide according to the manufacturer’s instructions and imaged in a clinical stimulated Raman scattering microscope (NIO Laser Imaging System, Invenio Imaging, Santa Clara, CA) [21].

## Results

**Fig. 2:**
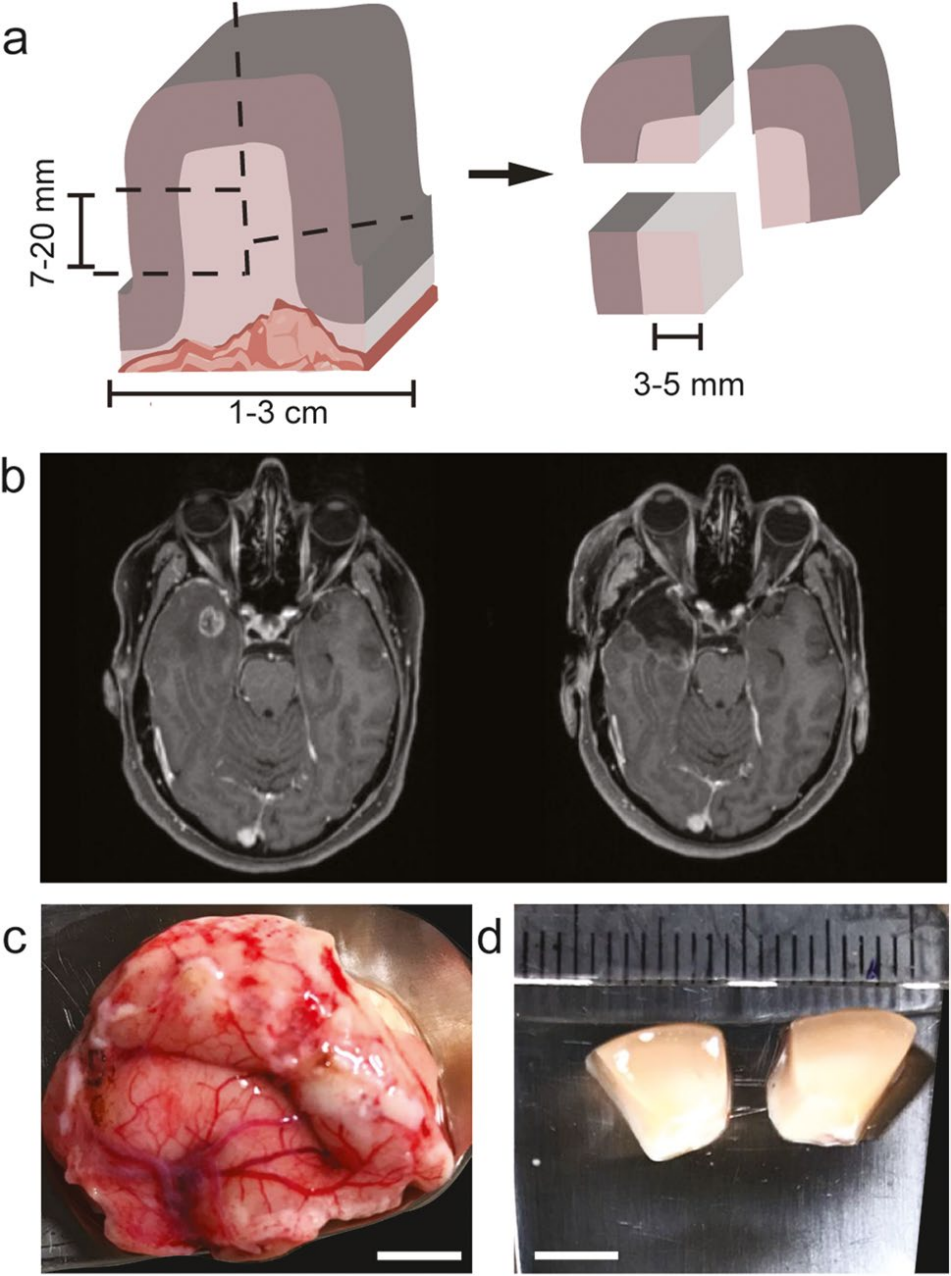
(**a**) Illustration of larger samples with potentially tumorous portions and the dissection that is required for further processing in the laboratory. (**b**–**d**) Example of larger access tissue sample resection. (**b**) Pre- and postoperative T1 contrast-enhanced MRI of a 60-year-old female patient who underwent primary resection of a right temporal glioblastoma. (**c**) A larger sample of access tissue microdissected intraoperatively and (**d**) blocks resulting from a subdivision in the laboratory. Note the selection of macroscopically non-infiltrated tissue. Scale bar 5 mm

In multiple rounds of experiments, we developed and refined a set of surgical principles (Table 1) for the removal of cortical access tissue for clinical and basic research. Depending on the experimental use case, we developed two intraoperative sampling strategies: Smaller single samples were used for electrophysiology and electron microscopy (5–7 mm, Fig. 1), and larger samples were subdivided in the laboratory for the cultivation of organotypic slice cultures and transcriptomic analysis (1–3 cm, Fig. 2).

**Table 1:** Principles of brain tissue microdissection

Principles	
1. Rapid preparation (<1 min) to minimize hypoxia 2. Maintain cortical axis, make incisions perpendicular to the pial surface 3. Minimize mechanical trauma to sample 4. Use a pointed scalpel blade with serrating movements to avoid tissue compression 5. Avoid cauterization and blunt preparation 6. Apply constant irrigation to maintain visibility and avoid blood clotting 7. Retrieve the sample without the use of forceps or suction

Multiple surgeons (*n* = 8) adopted the technique after a single round of explanation using the graphics shown in Fig. 1a–c. No adverse events from sample resection were observed, especially no bleeding complications. The duration of sample extraction ranged from 40 to 120 s and was ideally performed in less than 1 min.

**Fig. 3:**
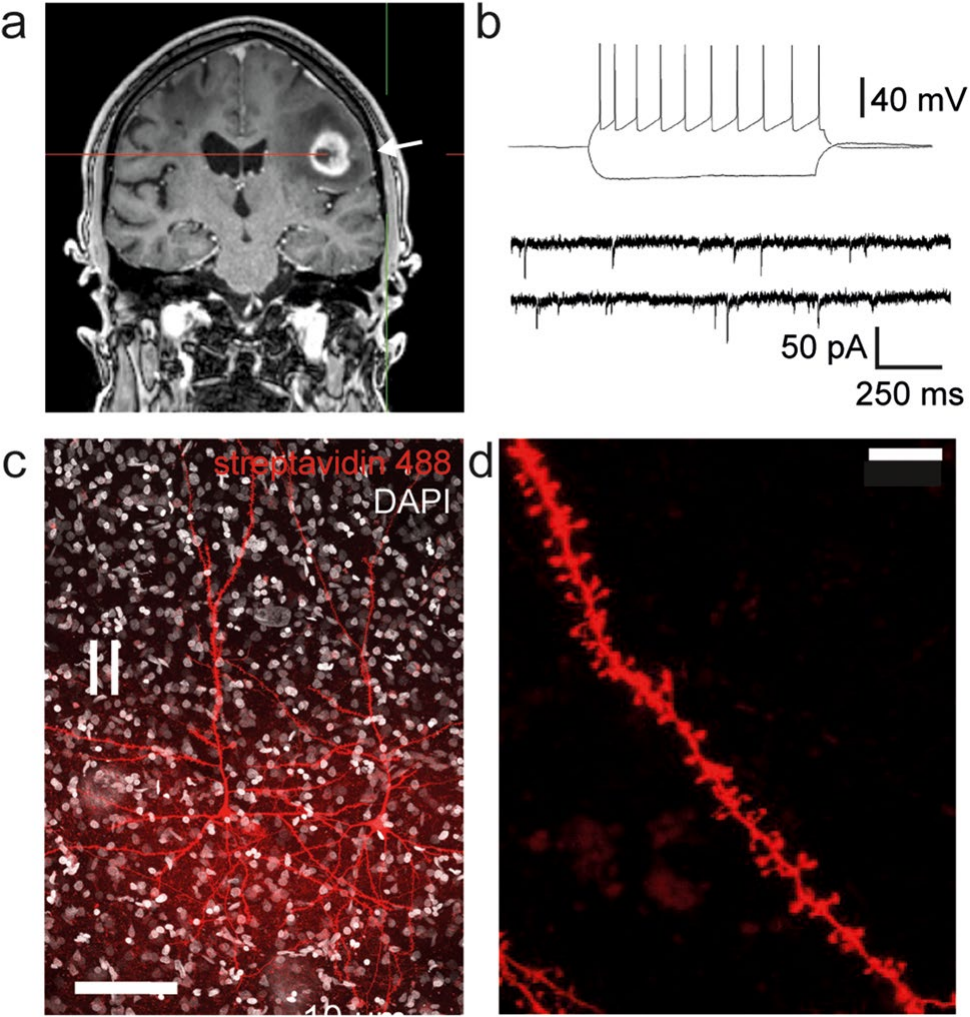
Illustration of Case 1: Exemplary electrophysiological recordings with subsequent post-hoc visualization. (**a**) The arrow indicates the location of the cortical access tissue sample (5–7 mm) in T1 contrast-enhanced MRI. (**b**) Firing pattern in response to step current injection and miniature excitatory postsynaptic currents of a layer 2/3 pyramidal neuron. (**c**) Post-hoc visualization of recorded layer 2/3 pyramidal neuron with (**d**) magnification of a basal dendrite with dendritic spines. Scale bar 100 μm and 10 μm

A prerequisite for standardization of the experimental settings is the maintenance of the cortical axis perpendicular to the pial surface already at the stage of the sample removal (Fig. 1b). The apical dendrites (Figs. 1b and 3c) of cortical pyramidal neurons are orientated along the perpendicular cortical axis. A transection of the apical dendrite of cortical neurons should be avoided to preserve electrophysiological properties [6, 13] and allow the identification of neurons. Deviations of the cortical axis during the intraoperative dissection require later adjustments representing a time delay, loss of tissue, and additional mechanical trauma.

### Preparation of single cortical samples

Rectangular incisions (~5–7 mm) were made perpendicular to the pial surface using a pointed scalpel (Fig. 1d). Incision should be placed at the top of gyri to avoid entering sulci where larger blood vessels are found to prevent bleeding complications. To avoid compression and shear of the tissue, serrating movements of the blade were applied (see Supplementary Video 1). A narrow dissector was introduced from one side, and the white matter under the sample was carefully dissected while causing minimal mechanical disturbance to the cortical part of the sample (Fig. 1c). It was helpful to angulate (~45°) (Fig. 1a) one side of the rectangular incision to allow the introduction of the dissector and minimize compression of the surrounding tissue during preparation. The tissue block was lifted using the dissector and flipped onto a brain spatula (Fig. 1c). The tissue and the brain spatula were immediately immersed into a non-sterile container filled with transportation media and removed from the operating field and transported to the internal (5 min) and external laboratory facilities (15 min).

This preparation technique is optimized for the preservation of superficial layer cortical neurons. Deep layers of neurons, as well as the underlying white matter, are at risk of mechanical damage. An optimally prepared single block of 5–7 mm size can immediately be processed in the vibratome (Supplementary Fig. 2), minimizing delay and mechanical damage. Without the need for further trimming, the slices can be cut at slightly higher temperatures (7–11 °C), reducing the effect of low temperatures on ultrastructure and electrophysiological properties [11]. Blood clots on the sample may lead to detachment during processing in the vibratome and are not easily removed once they are attached to the sample. Therefore, constant irrigation of the sample during surgery proved to be beneficial (Table 1).

Using this technique, 36 brain tissue samples, of which 8 were reported previously [15], were obtained from frontal (*n* = 14), parietal (*n* = 2), temporal (*n* = 16), and occipital lobe (*n* = 4). Twenty-nine samples were from tumors and 7 from epilepsy surgeries. Eight to ten brain slices can result from one single block of tissue. Under ideal conditions, up to 20–70 cells could be recorded from a single sample.

### Preparation of large samples

The same surgical steps as described above were applied for the microdissection of larger (10–30 mm) brain tissue samples. In larger samples, subcortical structures remained intact (Fig. 2). After transfer to the laboratory in preparation medium, larger samples were divided into blocks of various sizes according to the intended experimental use case (Table 2). Dissection was best performed in an ice-cold preparation medium, taking care to discard macroscopically infiltrated and tumorous tissue (Fig. 2a). For the generation of tumor invasion models [7], a certain portion (3–5 mm) of the white matter was preserved (Fig. 5). As previously shown in the literature [23], a single block of the size of 7–20 mm resulted in 18–20 organotypic slice models that could be cultivated for up to 12 days (*n* = 25).

**Table 2:** Overview of the sample size, medium, and tolerated delay (transport time) that need to be taken into account during surgery in order to apply various methods to human cortical access tissue

Method	Size [mm]	Transfer medium	Time [min]	Reference
Single sample				
Electrophysiology electron microscopy	5–7	NMDG aCSF	15	[15, 38]
Stimulated Raman histology	1–4	Moist gauze	20	[8, 21, 35]
Large sample				
Organotypic cell culture	7–20	Prep. medium	5	[7, 23, 30]
Spatial transcriptomics	6		5–10	[25, 33]
Single-cell transcriptomics	20–40		20	[24]
Bulk transcriptomics	1–2		5–20	[7]
Mass spectroscopy	1–2		5–10	[17]

### Preparation of samples for intraoperative stimulated Raman histology

Stimulated Raman histology (SRH) [8, 21, 22, 35] allows the intraoperative label-free histological evaluation of tumorous samples. These samples were the least sensitive to mechanical damage, as the preparation itself consisted of a squash preparation. However, cauterization and the use of ultrasonic aspirators (CUSA) should be avoided. We found an endoscopic biopsy forceps optimal for targeted sampling of tumor and infiltrated brain tissue [21] (Fig. 6b). Samples sized 1–4 mm were sufficient for SRH imaging (Fig. 6b, c). The ease of use and the standardized imaging conditions make intraoperative SRH also an ideal tool to assess the quality and tumor burden of unstained neocortical access tissue slices (Fig. 6e–g).

### Example cases

Case 1: Figure 3 shows a whole-cell patch-clamp recording of a superficial layer 2/3 pyramidal neuron from a 73-year-old male patient undergoing surgery for the resection of an IDH wild-type glioblastoma in the left frontal lobe (Figs. 1d and 3a, Supplementary Video 1). The action potential firing patterns in response to a current injection as well as spontaneous excitatory synaptic currents demonstrate the viability of the tissue (Fig. 3b). Two-layer 2/3 pyramidal neurons from the same sample were recorded and visualized with a streptavidin-coupled fluorescent labeling (Fig. 3c). The recordings were performed in a laboratory situated 10 min away from the neurosurgical operating room [15].

**Fig. 4:**
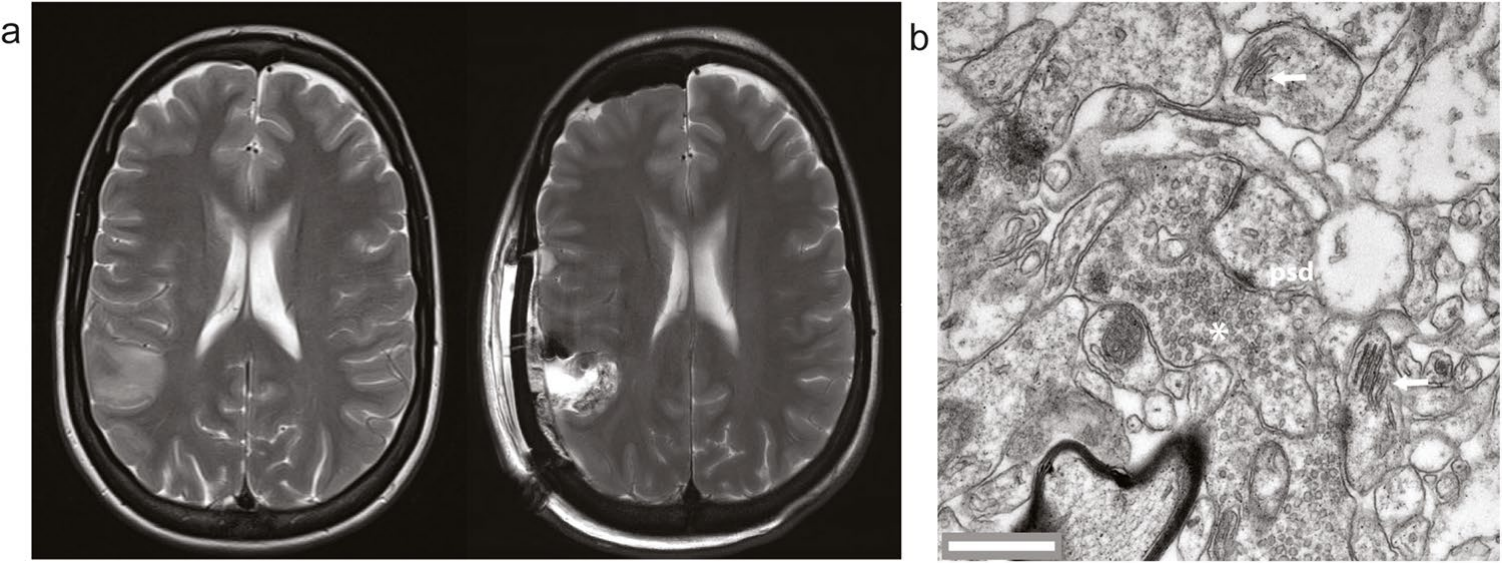
Illustration of Case 2: (**a**) Pre- and postoperative T2 weighted MRI. (**b**) Transmission electron micrograph of an acute slice of neocortical access tissue showing pre- and postsynaptic structures such as vesicles (*), postsynaptic density (psd), and the spine apparatus organelle (arrows). Scale bar 0.5 μm

Case 2: Figure 4a shows the case of a 19-year-old female patient who underwent the resection of a diffuse astrocytoma CNS-WHO grade 2 (IDH mutated) in the right supramarginal gyrus during awake surgery. Electron micrographs generated from an acute slice of a small block of the tissue showed a high quality of ultrastructural preservation that allows the analysis of synapses as well as intracellular organelles like the spine apparatus organelle (Fig. 4b) [29].

**Fig. 5:**
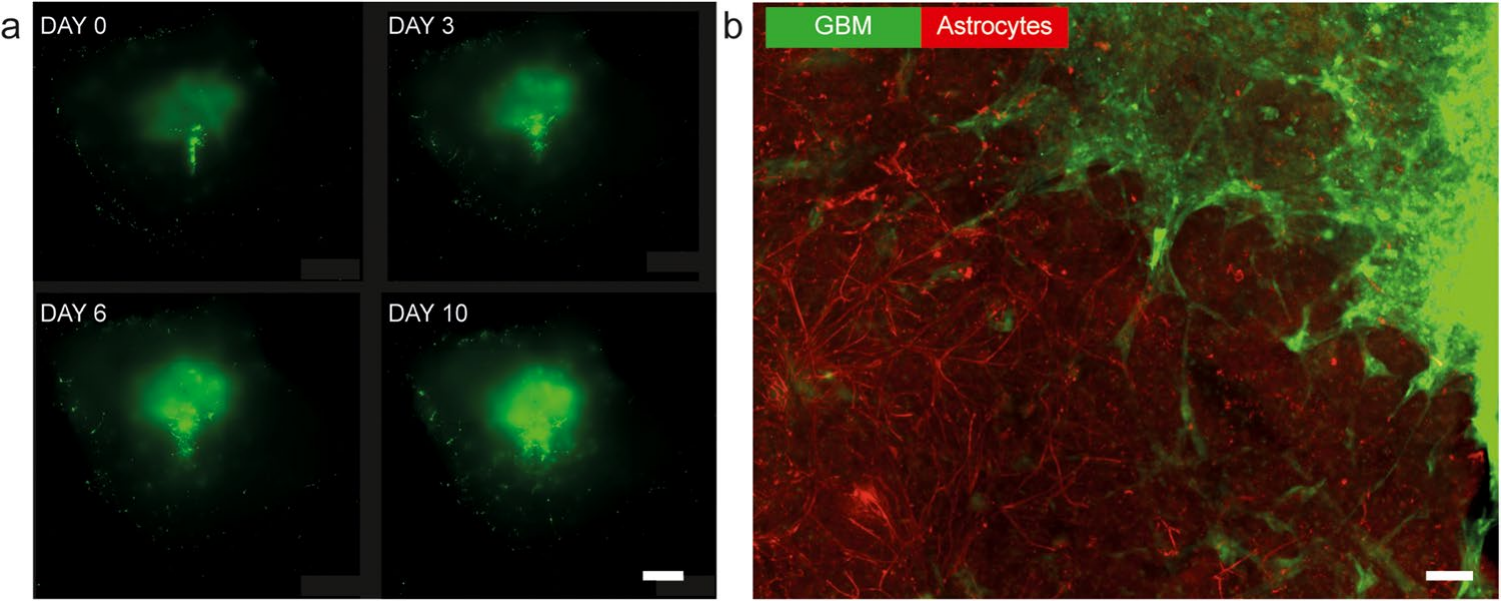
Illustration of Case 3: Ex vivo glioblastoma invasion model. (**a**) Repeated images of fluorescently labeled GBM cells (ZsGreen) that were injected into the white matter compartment of organotypic brain slice cultures generated from human cortical access tissue. Tissue was imaged for 10 days post-injection, scale bar 1 mm. (**b**) Fixation and immunofluorescent labeling reveals the interaction of tumor cells (zsGreen) with the astrocytes of the slice (red: GFAP, Alexa 555). Scale bar 50 μm

Case 3: Figure 5 shows an ex vivo glioblastoma invasion model based on organotypic slice cultures generated from the access tissue of a 59-year-old male patient who underwent surgery for the removal of a glioblastoma IDH wild-type in the right frontal lobe. The slice cultures were subcortically injected with fluorescently labeled GBM cells, and the growth was monitored for 10 days, highlighting the interaction with astrocytes of the slices.

**Fig. 6:**
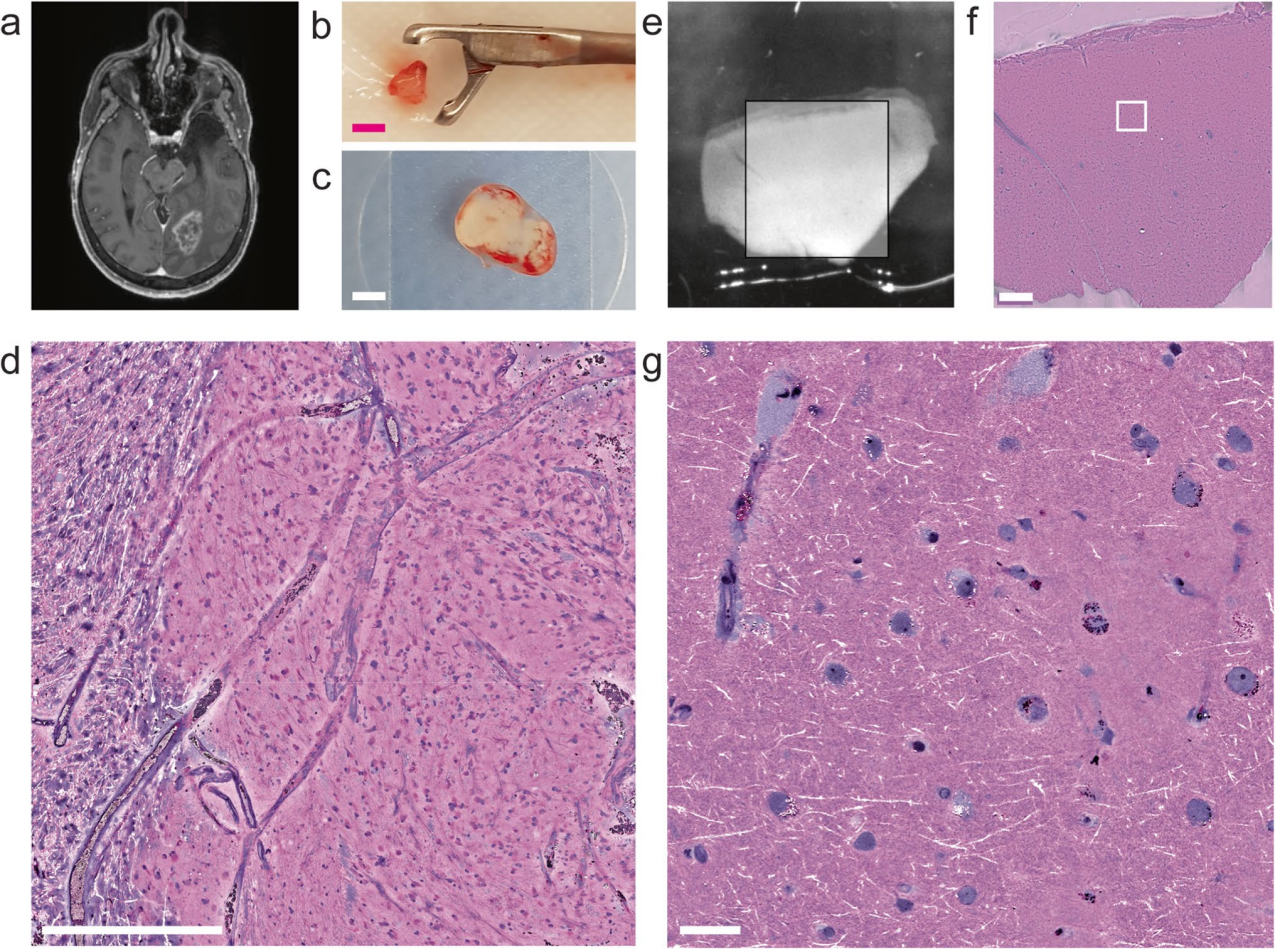
(**a**–**d**) Illustration of Case 4: (**a**) the arrow indicates the location of the tumor in T1 contrast-enhanced MRI. (**b**) Sample after removal using the endoscopic cutting forceps. Scale bar 2 mm. (**c**) The squash preparation of the same sample on the imaging slide. Scale bar 2 mm. (**d**) Exemplary image of SRH showing infiltrated white matter with degenerated myelinated axons (left) and blood vessels surrounding tumor cells (center). Scale bar 500 μm. (**e**–**g**) Illustration of Case 5: (**e**) 250-μm-thick slice of neocortical access tissue mounted on imaging slide. Box ~5 mm. (**f**) SRH image of the same neocortical slice. Scale bar 500 μm. (**g**) Magnification of image in (f) showing capillaries, myelinated axons, and neurons containing cytoplasmatic granula. Scale bar 50 μm

Case 4: Figure 6a–d shows the case of a 57-year-old female patient who underwent surgery for a recurrent CNS-WHO grade 4 astrocytoma (IDH mutated) in the left occipital lobe; 7 months prior to surgery, radiation therapy was performed. A 2–3 mm sample of the tumor was removed using biopsy forceps and stimulated Raman histology was performed in the OR.

Case 5: Figure 6e–g shows the neocortical access tissue of a 60-year-old female who underwent resection of a left temporal metastasis of an adenocarcinoma. Stimulated Raman histology was used to assess tissue quality and to rule out tumor infiltration in a slice of access tissue.

## Discussion

In this technical report, we describe a surgical technique for the safe removal of human cortical access tissue ideal for various applications in basic neuroscientific and clinical research. The technique can easily be integrated into routine neurosurgical procedures without the necessity for additional surgical instruments.

The neurosurgeon should be aware of the intended use of the tissue (Table 2) and lays the foundation for the experimental success by the recruitment of appropriate candidate patients and by choosing the appropriate microdissection strategy. By optimizing the size and orientation of the sample in the OR, additional time-consuming and traumatic handling of the sample in the laboratory can be avoided. Counterintuitively, we found that larger samples that require further trimming are not necessarily better for the experimental success of acute slice experiments. In our experience, optimal electrophysiological results can already be achieved with relatively small samples (5–7 mm, Fig. 1).

The location of the sample is determined on the basis of clinical findings intraoperatively. Clinical and basic research interests must not interfere with the decision about the surgical strategy, the surgical route or trajectory, or the location of the cortical access tissue. We suggest that the exact location of the cortical access should be documented using neuronavigation in every case. Brain tissue acquisition will cause only a minimal temporal delay in the course of the operation (<2 min).

### Ethics and biobanking

To ensure a consistent and ethically sensitive approach, in the absence of standard guidelines on this emerging field of translational research, the pre-, peri-, and postoperative procedures should be formalized in a research protocol and ethics approval for this protocol should be sought by the local research ethics committee. To ensure consistency in the ethical standards for such procedures, an international and participatory consensus process should be initiated, e.g., organized by professional neurosurgery associations.

The technique outlined here allows to maximize the scientific gain of every cortical and subcortical tissue donation, leading to fewer sample numbers required to answer a range of questions in translational neuroscience. The combination of several techniques maximized the use of each individual sample. We encourage to set up an experimental protocol and biobanking infrastructure that allows the exchange of tissue and data among research institutions. With emerging technologies, the benefit of analyzing human tissue with a multitude of technologies is paramount.

### Standardization of tissue sampling

Human cortical access tissue has to be considered pathologically altered. The alterations arise from sample removal, sample processing [11], and the pathology (tumor, epilepsy) leading to the operation. The effects of the underlying pathology on cortical access tissue may range from tissue edema and histopathologically diagnosable tumor infiltration to subtle changes in the ultrastructure [5].

The macroscopic pathological effect can be controlled by measuring the distance of the cortical access tissue to the pathologies (contrast-enhancing border or FLAIR signal) [23] and microscopically using histological methods (e.g., immuno/H&E stain, stimulated Raman histology [22, 26, 35]) to assess tumor infiltration (Fig. 6e–f). Nonetheless, subtle changes will most likely only be discernible by exploration of the electrophysiological [9] and ultrastructural properties [16] across different samples, under the condition that the artifacts introduced by tissue microdissection and processing are reduced to a minimum.

### Limitations

The resection of neocortical tissue blocks and subsequent slice preparations involve a severing of long range and local axons as well as dendrites, thus preserving only parts of the neocortical circuitry [18]. Subcortical neuromodulatory inputs, which are essential for physiological function in vivo, will not be preserved ex vivo, a fact that has to be taken into consideration in the experimental design. In animal models, a solution to overcome the limitation of acute slice preparations includes a combination of in vivo recordings and post-hoc morphological reconstructions across entire brains [20, 28].

### Future outlook

A standardized sampling technique that reliably produces high-quality human brain tissue specimens forms the basis for the investigation of unique neurophysiological features in individual patients. Such characteristics can, for instance, include comorbidities such as neurodevelopmental and neurodegenerative disorders that can lead to or arise from alterations of neuronal wiring and plasticity [39, 43], or the intake of pharmacological agents. Furthermore, it will be possible to study the effect of interventions on the human brain, such as invasive and non-invasive brain stimulation techniques (e.g., TMS [4]), radiation [27], or tumor-treating fields [36]. A standardized sampling technique is therefore of utmost importance for current and future basic and clinical research and treatment development.

Improved tissue quality will also have an impact on pathological diagnosis. Although neuropathological diagnostic methods are moving toward molecular diagnostics that are also performed with lower-quality tissue [3], the interpretation of tumor infiltration in intact neuropil might greatly benefit from optimally preserved tissue. Further novel intraoperative imaging and spectroscopic techniques (SRH, FTIR) [8, 21, 22, 26, 34] that permit close to real-time histological examination of brain tissue in the operating room (Fig. 6) may influence surgical decision-making in the future [35]. Thus, optimally preserved tissue is essential for the validation of anatomical, molecular, and physiological findings of methods for intraoperative tissue analysis.

## Conclusion

The surgical extraction of human brain tissue lays the foundation for translational neuroscientific research on vital human brain tissue. The microdissection technique of human cortical access tissue is safe and easily adoptable to routine neurosurgical procedures. Even small samples of a few millimeters in size can be successfully used for electrophysiological experiments. Standardized techniques of sample extraction greatly improve the success rate and the reliability of results obtained from intraoperative human brain tissue specimens.

## Supplementary information

ESM 1 Suppl. Figure 1. Box for the transport of human brain samples under constant carbogenation (95% O_2_ 5% CO_2_) built from standard clinical supplies. (TIF 8626 kb)

High resolution image (PNG 1254 kb)

ESM 2 Suppl. Figure 2. Single 5-7 mm sample of human cortical access tissue prepared according to the schematic in Fig. 1 that can be immediately processed in a vibratome. Acute 300-400 μm thick slices can then be analyzed using electrophysiology, light- and electron microscopy. (TIF 5937 kb)

High resolution image (PNG 1146 kb)

ESM 3 Suppl. Video 1. Supplementary Video 1 shows the microdissection of the sample depicted in Fig. 1d. (MP4 34529 kb)

## Data Availability

Not applicable.

## Abbreviations

CNS: central nervous system
GBM: Glioblastoma
OR: Operating room
SRH: Stimulated Raman histology

## References

[1] Andersson M, Avaliani N, Svensson A, Wickham J, Pinborg LH, Jespersen B, Christiansen SH, Bengzon J, Woldbye DPD, Kokaia M (2016). Optogenetic control of human neurons in organotypic brain cultures. Sci Rep.

[2] Campagnola L, Seeman SC, Chartrand T, Kim L, Hoggarth A, Gamlin C, Ito S, Trinh J, Davoudian P, Radaelli C, Kim M-H, Hage T, Braun T, Alfiler L, Andrade J, Bohn P, Dalley R, Henry A, Kebede S et al Local connectivity and synaptic dynamics in mouse and human neocortex. Science 375:eabj5861. 10.1126/science.abj5861 10.1126/science.abj5861 PMC9970277 35271334

[3] Capper D, Jones DTW, Sill M, Hovestadt V, Schrimpf D, Sturm D, Koelsche C, Sahm F, Chavez L, Reuss DE, Kratz A, Wefers AK, Huang K, Pajtler KW, Schweizer L, Stichel D, Olar A, Engel NW, Lindenberg K, Harter PN, Braczynski AK, Plate KH, Dohmen H, Garvalov BK, Coras R, Hölsken A, Hewer E, Bewerunge-Hudler M, Schick M, Fischer R, Beschorner R, Schittenhelm J, Staszewski O, Wani K, Varlet P, Pages M, Temming P, Lohmann D, Selt F, Witt H, Milde T, Witt O, Aronica E, Giangaspero F, Rushing E, Scheurlen W, Geisenberger C, Rodriguez FJ, Becker A, Preusser M, Haberler C, Bjerkvig R, Cryan J, Farrell M, Deckert M, Hench J, Frank S, Serrano J, Kannan K, Tsirigos A, Brück W, Hofer S, Brehmer S, Seiz-Rosenhagen M, Hänggi D, Hans V, Rozsnoki S, Hansford JR, Kohlhof P, Kristensen BW, Lechner M, Lopes B, Mawrin C, Ketter R, Kulozik A, Khatib Z, Heppner F, Koch A, Jouvet A, Keohane C, Mühleisen H, Mueller W, Pohl U, Prinz M, Benner A, Zapatka M, Gottardo NG, Driever PH, Kramm CM, Müller HL, Rutkowski S, von Hoff K, Frühwald MC, Gnekow A, Fleischhack G, Tippelt S, Calaminus G, Monoranu C-M, Perry A, Jones C, Jacques TS, Radlwimmer B, Gessi M, Pietsch T, Schramm J, Schackert G, Westphal M, Reifenberger G, Wesseling P, Weller M, Collins VP, Blümcke I, Bendszus M, Debus J, Huang A, Jabado N, Northcott PA, Paulus W, Gajjar A, Robinson GW, Taylor MD, Jaunmuktane Z, Ryzhova M, Platten M, Unterberg A, Wick W, Karajannis MA, Mittelbronn M, Acker T, Hartmann C, Aldape K, Schüller U, Buslei R, Lichter P, Kool M, Herold-Mende C, Ellison DW, Hasselblatt M, Snuderl M, Brandner S, Korshunov A, von Deimling A, Pfister SM (2018). DNA methylation-based classification of central nervous system tumours. Nature.

[4] Duffau H (2020). Functional mapping before and after low-grade glioma surgery: a new way to decipher various spatiotemporal patterns of individual neuroplastic potential in brain tumor patients. Cancers.

[5] Fiala JC, Spacek J, Harris KM (2002). Dendritic spine pathology: cause or consequence of neurological disorders?. Brain Res Rev.

[6] Gidon A, Zolnik TA, Fidzinski P, Bolduan F, Papoutsi A, Poirazi P, Holtkamp M, Vida I, Larkum ME (2020). Dendritic action potentials and computation in human layer 2/3 cortical neurons. Science.

[7] Henrik Heiland D, Ravi VM, Behringer SP, Frenking JH, Wurm J, Joseph K, Garrelfs NWC, Strähle J, Heynckes S, Grauvogel J, Franco P, Mader I, Schneider M, Potthoff A-L, Delev D, Hofmann UG, Fung C, Beck J, Sankowski R, Prinz M, Schnell O (2019). Tumor-associated reactive astrocytes aid the evolution of immunosuppressive environment in glioblastoma. Nat Commun.

[8] Hollon TC, Pandian B, Adapa AR, Urias E, Save AV, Khalsa SSS, Eichberg DG, D’Amico RS, Farooq ZU, Lewis S, Petridis PD, Marie T, Shah AH, Garton HJL, Maher CO, Heth JA, McKean EL, Sullivan SE, Hervey-Jumper SL, Patil PG, Thompson BG, Sagher O, McKhann GM, Komotar RJ, Ivan ME, Snuderl M, Otten ML, Johnson TD, Sisti MB, Bruce JN, Muraszko KM, Trautman J, Freudiger CW, Canoll P, Lee H, Camelo-Piragua S, Orringer DA (2020). Near real-time intraoperative brain tumor diagnosis using stimulated Raman histology and deep neural networks. Nat Med.

[9] Jones RSG, da Silva AB, Whittaker RG, Woodhall GL, Cunningham MO (2016). Human brain slices for epilepsy research: pitfalls, solutions and future challenges. J Neurosci Methods.

[10] Kato H, Ito Z, Matsuoka S, Sakurai Y (1973). Electrical activities of neurons in the sliced human cortex in vitro. Electroencephalogr Clin Neurophysiol.

[11] Kirov SA, Petrak LJ, Fiala JC, Harris KM (2004). Dendritic spines disappear with chilling but proliferate excessively upon rewarming of mature hippocampus. Neuroscience.

[12] Kovacs FE, Knop T, Urbanski MJ, Freiman I, Freiman TM, Feuerstein TJ, Zentner J, Szabo B (2012). Exogenous and endogenous cannabinoids suppress inhibitory neurotransmission in the human neocortex. Neuropsychopharmacology.

[13] Larkum ME, Waters J, Sakmann B, Helmchen F (2007). Dendritic spikes in apical dendrites of neocortical layer 2/3 pyramidal neurons. J Neurosci.

[14] Le Duigou C, Savary E, Morin-Brureau M, Gomez-Dominguez D, Sobczyk A, Chali F, Milior G, Kraus L, Meier JC, Kullmann DM (2018). Imaging pathological activities of human brain tissue in organotypic culture. J Neurosci Methods.

[15] Lenz M, Kruse P, Eichler A, Straehle J, Beck J, Deller T, Vlachos A (2021). All-trans retinoic acid induces synaptic plasticity in human cortical neurons. Elife.

[16] Loomba S, Straehle J, Gangadharan V, Heike N, Khalifa A, Motta A, Ju N, Sievers M, Gempt J, Meyer HS, Helmstaedter M (2022). Connectomic comparison of mouse and human cortex. Science.

[17] Maier JP, Ravi VM, Kueckelhaus J, Behringer SP, Garrelfs N, Will P, Sun N, von Ehr J, Goeldner JM, Pfeifer D, Follo M, Hannibal L, Walch AK, Hofmann UG, Beck J, Heiland DH, Schnell O, Joseph K (2021). Inhibition of metabotropic glutamate receptor III facilitates sensitization to alkylating chemotherapeutics in glioblastoma. Cell Death Dis.

[18] Mohan H, Verhoog MB, Doreswamy KK, Eyal G, Aardse R, Lodder BN, Goriounova NA, Asamoah B, Brakspear ABC, Groot C, van der Sluis S, Testa-Silva G, Obermayer J, ZSRM B, Narayanan RT, Baayen JC, Segev I, Mansvelder HD, de Kock CPJ (2015). Dendritic and axonal architecture of individual pyramidal neurons across layers of adult human neocortex. Cereb Cortex.

[19] Molnár G, Oláh S, Komlósi G, Füle M, Szabadics J, Varga C, Barzó P, Tamás G (2008). Complex events initiated by individual spikes in the human cerebral cortex. PLoS Biol.

[20] Narayanan RT, Mohan H, Broersen R, de Haan R, Pieneman AW, de Kock CP (2014). Juxtasomal biocytin labeling to study the structure-function relationship of individual cortical neurons. J Vis Exp.

[21] Neidert N, Straehle J, Erny D, Sacalean V, El Rahal A, Steybe D, Schmelzeisen R, Vlachos A, Reinacher PC, Coenen VA, Mizaikoff B, Heiland DH, Prinz M, Beck J, Schnell O (2022). Stimulated Raman histology in the neurosurgical workflow of a major European neurosurgical center — part A. Neurosurg Rev.

[22] Orringer DA, Pandian B, Niknafs YS, Hollon TC, Boyle J, Lewis S, Garrard M, Hervey-Jumper SL, Garton HJL, Maher CO, Heth JA, Sagher O, Wilkinson DA, Snuderl M, Venneti S, Ramkissoon SH, McFadden KA, Fisher-Hubbard A, Lieberman AP, Johnson TD, Xie XS, Trautman JK, Freudiger CW, Camelo-Piragua S (2017). Rapid intraoperative histology of unprocessed surgical specimens via fibre-laser-based stimulated Raman scattering microscopy. Nat Biomed Eng.

[23] Ravi VM, Joseph K, Wurm J, Behringer S, Garrelfs N, d’Errico P, Naseri Y, Franco P, Meyer-Luehmann M, Sankowski R, Shah MJ, Mader I, Delev D, Follo M, Beck J, Schnell O, Hofmann UG, Heiland DH (2019). Human organotypic brain slice culture: a novel framework for environmental research in neuro-oncology. Life Sci Alliance.

[24] Ravi VM, Neidert N, Will P, Joseph K, Maier JP, Kückelhaus J, Vollmer L, Goeldner JM, Behringer SP, Scherer F, Boerries M, Follo M, Weiss T, Delev D, Kernbach J, Franco P, Schallner N, Dierks C, Carro MS, Hofmann UG, Fung C, Sankowski R, Prinz M, Beck J, Salié H, Bengsch B, Schnell O, Heiland DH (2022). T-cell dysfunction in the glioblastoma microenvironment is mediated by myeloid cells releasing interleukin-10. Nat Commun.

[25] Ravi VM, Will P, Kueckelhaus J, Sun N, Joseph K, Salié H, Vollmer L, Kuliesiute U, von Ehr J, Benotmane JK, Neidert N, Follo M, Scherer F, Goeldner JM, Behringer SP, Franco P, Khiat M, Zhang J, Hofmann UG, Fung C, Ricklefs FL, Lamszus K, Boerries M, Ku M, Beck J, Sankowski R, Schwabenland M, Prinz M, Schüller U, Killmer S, Bengsch B, Walch AK, Delev D, Schnell O, Heiland DH (2022). Spatially resolved multi-omics deciphers bidirectional tumor-host interdependence in glioblastoma. Cancer Cell.

[26] Reinecke D, von Spreckelsen N, Mawrin C, Ion-Margineanu A, Fürtjes G, Jünger ST, Khalid F, Freudiger CW, Timmer M, Ruge MI, Goldbrunner R, Neuschmelting V (2022). Novel rapid intraoperative qualitative tumor detection by a residual convolutional neural network using label-free stimulated Raman scattering microscopy. Acta Neuropathol Commun.

[27] Robbins M, Greene-Schloesser D, Peiffer A, Shaw E, Chan M, Wheeler K (2012). Radiation-induced brain injury: a review. Front Oncol.

[28] Rojas-Piloni G, Guest JM, Egger R, Johnson AS, Sakmann B, Oberlaender M (2017). Relationships between structure, in vivo function and long-range axonal target of cortical pyramidal tract neurons. Nat Commun.

[29] Rosado J, Bui VD, Haas CA, Beck J, Queisser G, Vlachos A (2022). Calcium modeling of spine apparatus-containing human dendritic spines demonstrates an “all-or-nothing” communication switch between the spine head and dendrite. PLoS Comput Biol.

[30] Schneider M, Vollmer L, Potthoff A-L, Ravi VM, Evert BO, Rahman MA, Sarowar S, Kueckelhaus J, Will P, Zurhorst D (2021) Meclofenamate causes loss of cellular tethering and decoupling of functional networks in glioblastoma. Neuro-oncology 10.1093/neuonc/noab092 PMC8563322 33864086

[31] Schwarz N, Uysal B, Welzer M, Bahr JC, Layer N, Löffler H, Stanaitis K, Pa H, Weber YG, Hedrich UB, Honegger JB, Skodras A, Becker AJ, Wuttke TV, Koch H (2019). Long-term adult human brain slice cultures as a model system to study human CNS circuitry and disease. eLife.

[32] Shapson-Coe A, Januszewski M, Berger DR, Pope A, Wu Y, Blakely T, Schalek RL, Li PH, Wang S, Maitin-Shepard J, Karlupia N, Dorkenwald S, Sjostedt E, Leavitt L, Lee D, Bailey L, Fitzmaurice A, Kar R, Field B, Wu H, Wagner-Carena J, Aley D, Lau J, Lin Z, Wei D, Pfister H, Peleg A, Jain V, Lichtman JW (2021). A connectomic study of a petascale fragment of human cerebral cortex.

[33] Ståhl PL, Fredrik S, Sanja V, Anna L, Fernández NJ, Jens M, Stefania G, Michaela A, Westholm JO, Mikael H, Annelie M, Sten L, Simone C, Åke B, Fredrik P, Igor CP, Pelin S, Jan M, Olaf B, Joakim L, Jonas F (2016). Visualization and analysis of gene expression in tissue sections by spatial transcriptomics. Science.

[34] Steiner G, Galli R, Preusse G, Michen S, Meinhardt M, Temme A, Sobottka SB, Juratli TA, Koch E, Schackert G, Kirsch M, Uckermann O (2022) A new approach for clinical translation of infrared spectroscopy: exploitation of the signature of glioblastoma for general brain tumor recognition. J Neurooncol. 10.1007/s11060-022-04204-3 10.1007/s11060-022-04204-3 PMC9886632 36509907

[35] Straehle J, Erny D, Neidert N, Heiland DH, El Rahal A, Sacalean V, Steybe D, Schmelzeisen R, Vlachos A, Mizaikoff B, Reinacher PC, Coenen VA, Prinz M, Beck J, Schnell O (2022). Neuropathological interpretation of stimulated Raman histology images of brain and spine tumors: part B. Neurosurg Rev.

[36] Stupp R, Taillibert S, Kanner A, Read W, Steinberg DM, Lhermitte B, Toms S, Idbaih A, Ahluwalia MS, Fink K, Di Meco F, Lieberman F, Zhu J-J, Stragliotto G, Tran DD, Brem S, Hottinger AF, Kirson ED, Lavy-Shahaf G, Weinberg U, Kim C-Y, Paek S-H, Nicholas G, Bruna J, Hirte H, Weller M, Palti Y, Hegi ME, Ram Z (2017). Effect of tumor-treating fields plus maintenance temozolomide vs maintenance temozolomide alone on survival in patients with glioblastoma: a randomized clinical trial. JAMA.

[37] Ting JT, Kalmbach B, Chong P, Frates R, Keene CD, Gwinn RP, Cobbs C, Ko AL, Ojemann JG, Ellenbogen RG (2018). A robust ex vivo experimental platform for molecular-genetic dissection of adult human neocortical cell types and circuits. Sci Rep.

[38] Ting JT, Lee BR, Chong P, Soler-Llavina G, Cobbs C, Koch C, Zeng H, Lein E (2018). Preparation of acute brain slices using an optimized N-methyl-D-glucamine protective recovery method. J Vis Exp.

[39] Toga AW, Thompson PM (2014). Connectopathy in ageing and dementia. Brain.

[40] Verhoog MB, Goriounova NA, Obermayer J, Stroeder J, Hjorth JJJ, Testa-Silva G, Baayen JC, de Kock CPJ, Meredith RM, Mansvelder HD (2013). Mechanisms underlying the rules for associative plasticity at adult human neocortical synapses. J Neurosci.

[41] Witcher MR, Park YD, Lee MR, Sharma S, Harris KM, Kirov SA (2010). Three-dimensional relationships between perisynaptic astroglia and human hippocampal synapses. Glia.

[42] Yakoubi R, Rollenhagen A, von Lehe M, Shao Y, Sätzler K, Lübke JHR (2019). Quantitative three-dimensional reconstructions of excitatory synaptic boutons in layer 5 of the adult human temporal lobe neocortex: a fine-scale electron microscopic analysis. Cereb Cortex.

[43] Yamasaki T, Maekawa T, Fujita T, Tobimatsu S (2017). Connectopathy in autism spectrum disorders: a review of evidence from visual evoked potentials and diffusion magnetic resonance imaging. Front Neurosci.

