# Supplementary figures and images for: Technical report: surgical preparation of human brain tissue for clinical and basic research
Source: Acta Neurochir (Wien). 2023 May 6;165(6):1461–71. doi: 10.1007/s00701-023-05611-9 (PMC10227129; doi:10.1007/s00701-023-05611-9)

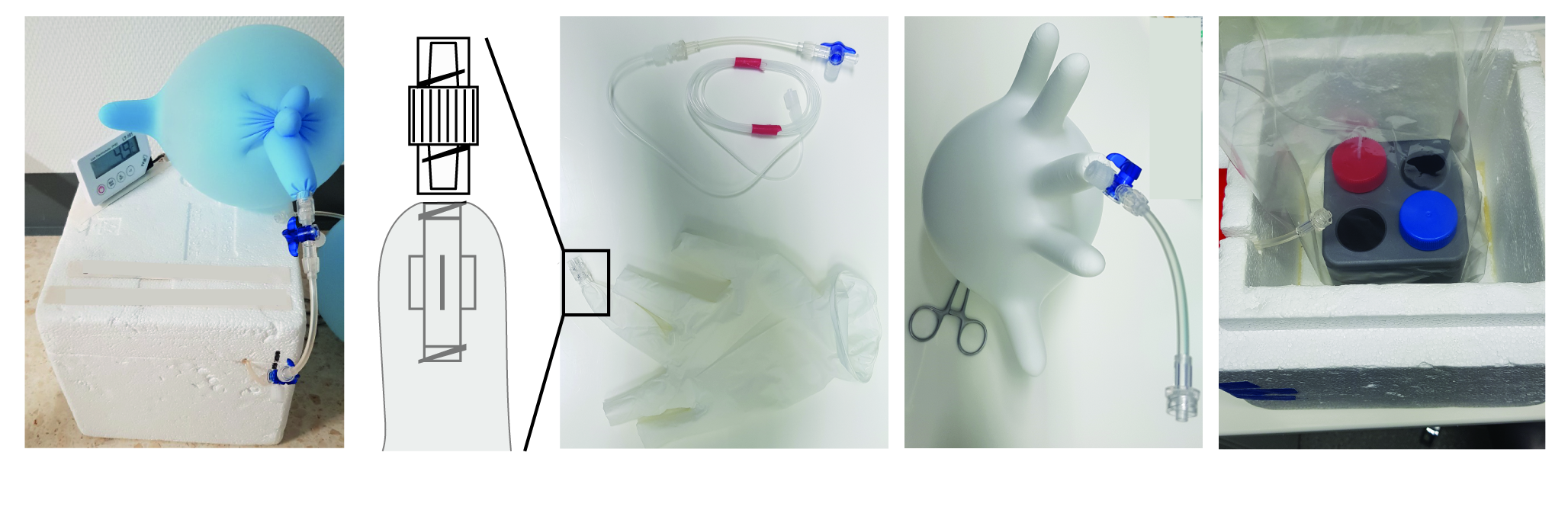

Supplement: Supplementary file 1 — Suppl. Figure 1. Box for the transport of human brain samples under constant carbogenation (95% O2 5% CO2) built from standard clinical supplies. (TIF 8626 kb) [file 701_2023_5611_MOESM1_ESM.tif]

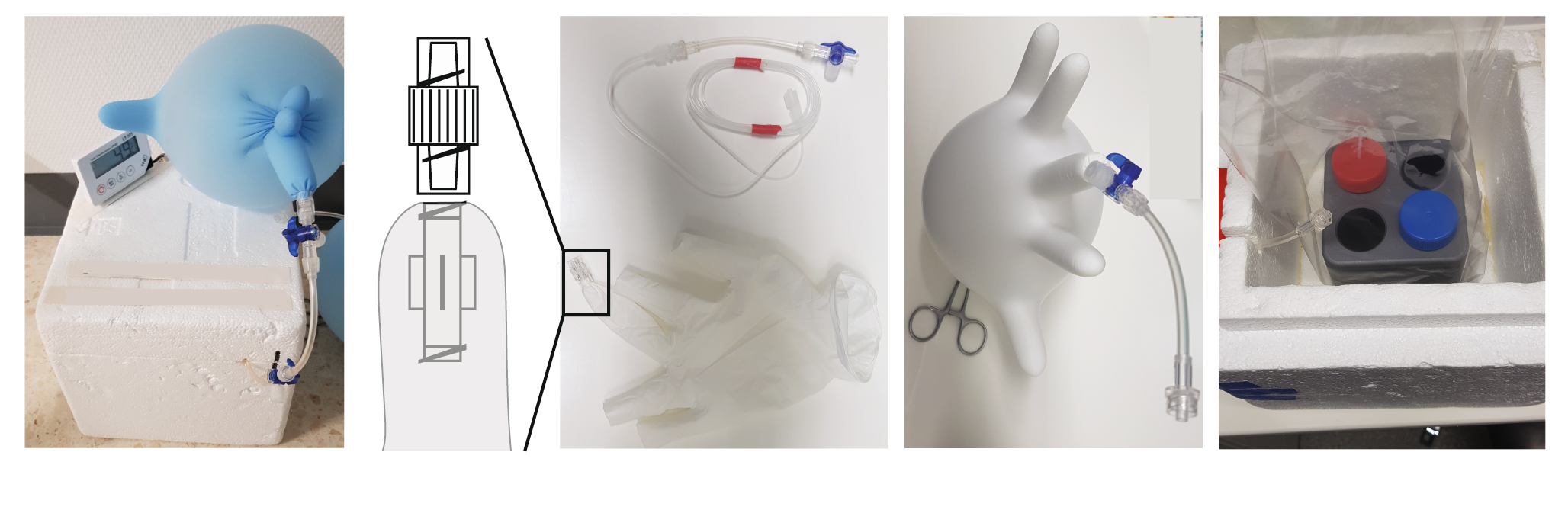

Supplement: Supplementary file 2 — High resolution image (PNG 1254 kb) [file 701_2023_5611_Fig7_ESM.png]

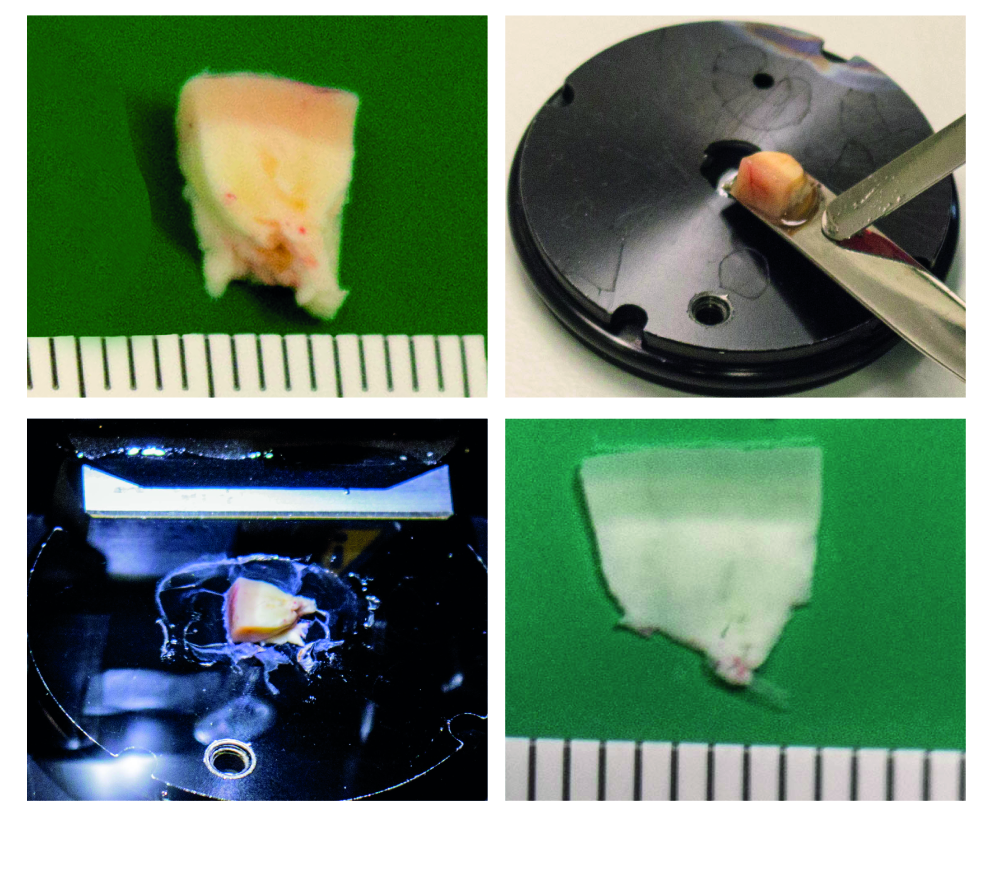

Supplement: Supplementary file 3 — Suppl. Figure 2. Single 5-7 mm sample of human cortical access tissue prepared according to the schematic in Fig. 1 that can be immediately processed in a vibratome. Acute 300-400 μm thick slices can then be analyzed using electrophysiology, light- and electron microscopy. (TIF 5937 kb) [file 701_2023_5611_MOESM2_ESM.tif]

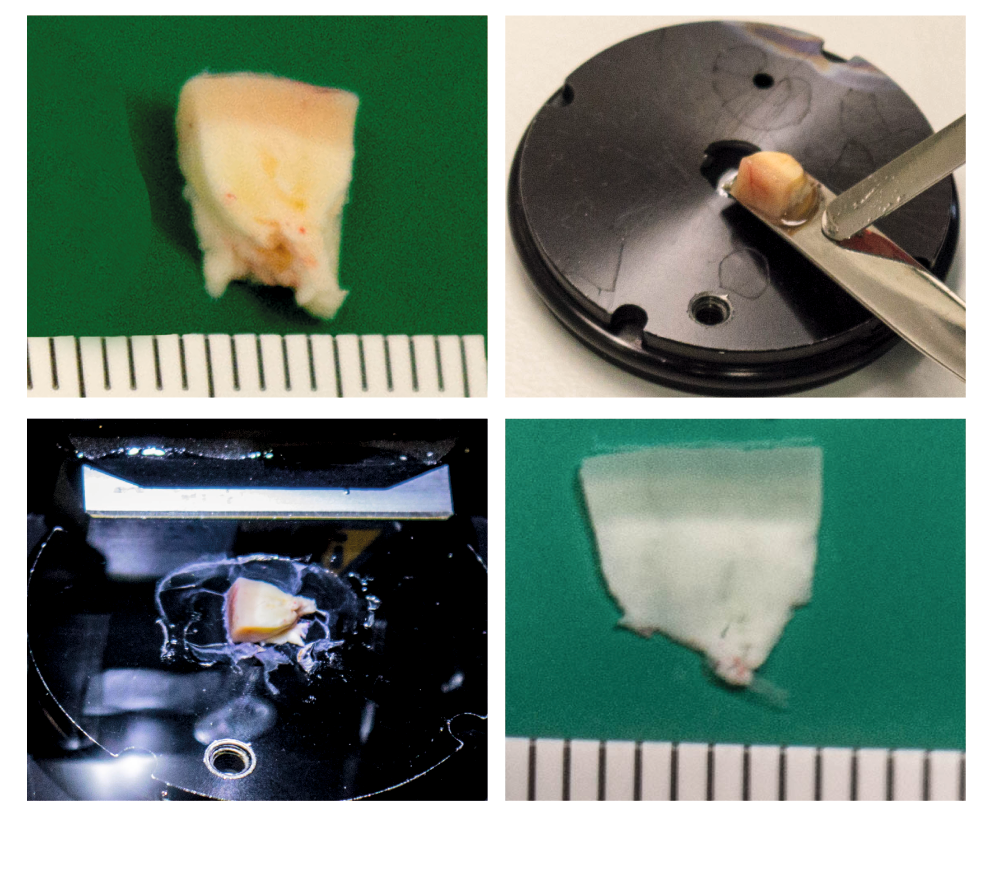

Supplement: Supplementary file 4 — High resolution image (PNG 1146 kb) [file 701_2023_5611_Fig8_ESM.png]
